# Hand hygiene and glove use in nursing homes before and after an intervention

**DOI:** 10.1017/ice.2020.1415

**Published:** 2021-02-09

**Authors:** Gwen R. Teesing, Jan Hendrik Richardus, Vicki Erasmus, Mariska Petrignani, Marion P. G. Koopmans, Margreet C. Vos, Jos M.G.A. Schols, Helene A.C.M. Voeten

**Affiliations:** 1 Department of Public Health, Erasmus MC, University Medical Center Rotterdam, Rotterdam, The Netherlands; 2 Municipal Public Health Service Rotterdam-Rijnmond, Rotterdam, The Netherlands; 3 Municipal Public Health Service Amsterdam, Amsterdam, The Netherlands; 4 Viroscience Department, Erasmus MC, University Medical Center Rotterdam, Rotterdam, The Netherlands; 5 Department of Medical Microbiology and Infectious Diseases, Erasmus MC, University Medical Center Rotterdam, Rotterdam, The Netherlands; 6 Department Health Services Research, CAPHRI, Maastricht University, Maastricht, The Netherlands

## Abstract

We investigated whether an intervention to improve hand hygiene compliance in nursing homes changed glove use. Hand hygiene compliance increased, but substitution of hand hygiene with gloves did not decrease. We observed a reduction of inappropriately unchanged gloves after exposure to body fluids.

Clinical trials identifier: Netherlands Trial Register, trial NL6049 (NTR6188): https://www.trialregister.nl/trial/6049.

Hand hygiene (HH) is a cornerstone of infection prevention programs in nursing homes. Yet, HH is often lacking when gloves are donned or doffed.^1^ Although gloves are necessary before a sterile procedure, when a healthcare worker (HCW) expects contact with body fluids, and when using contact precautions, gloves should be used in combination with HH.^2^ HH is necessary before donning gloves because micro-organisms on hands can contaminate the outsides of gloves (and other gloves in the same box). HH is also necessary after removing gloves, since microorganisms on gloves can contaminate hands and wrists during glove removal.

When an HCW dons or doffs gloves at an HH opportunity without performing HH, we assume that the HCW knows that an infection prevention activity should be done. We therefore consider this replacing HH by glove use (ie, ‘substitution’). Being unaware of the importance of the WHO guidelines and suboptimal availability of HH materials has been shown to cause low compliance with HH and glove protocol.^3^

The primary goal of this paper is to investigate whether the HH intervention in the HANDSOME study decreased substitution of HH by glove use. We also explore other glove use at HH opportunities.

## Methods

In this before-and-after study, we used data from a cluster randomized controlled trial to evaluate an HH intervention (HANDSOME study). The protocol and HH compliance outcomes are described elsewhere.^4,5^ The present study analyses glove use in the intervention arm of the trial.

### Definitions and data collection

All HH opportunities were registered in accordance with the WHO-defined HH moments.^6^ Total HH compliance rates exclude food- and medication-related opportunities. HH was defined as compliant if the nurse used either alcohol-based hand rub (ABHR) or the combination of soap, water, and a paper towel at a WHO-defined HH opportunity, regardless of glove use. HH compliance was measured through unobtrusive direct observation at baseline (October 2016) and follow-up (4 months, 7 months, and 1 year after the baseline observation). We recorded whether HH was performed, which WHO-defined moment it was, which submoment (when applicable), and glove use. Gloves were considered inappropriately unchanged if the nurse was wearing the same gloves as during a previous activity (moments 1 or 2) or if the nurse did not remove gloves after an activity for which HH was indicated (moments 3, 4, and 5). No distinction was made between sterile and nonsterile gloves.

### Analysis

At every HH opportunity, the nurse could do one of the following actions: (1) perform HH and not use gloves, (2) perform HH and don and doff gloves, (3) perform no HH, but don and doff gloves (substitution), (4) perform no HH and inappropriately not change gloves, or (5) perform no HH and not wear gloves. The rate of each category was calculated as the number of times that the action occurred, divided by the total number of WHO-defined HH opportunities, expressed as a percentage. We recorded frequently occurring submoments, specifically (1) before or after washing and/or perineal care in own room, (2) before or after helping at the toilet, (3) after an aseptic procedure, and (4) after removing bedding. Differences in glove-related behavior between baseline and follow-up measurements were statistically tested in multilevel analyses, controlling for the clustering of observations within nursing homes and nurses. Because differences are easily statistically significant due to the large number of observed HH opportunities, we considered them to be relevant (and presented the statistical test results) when there was an absolute difference of at least 10%. We also investigated the actions per observed nurse in multilevel analyses, controlling for clustering of observations within nursing homes. Nurses were included if they were observed for 5 or more HH opportunities. Odds ratios (OR) were calculated with 95% confidence intervals (CIs). All data were analyzed using IBM SPSS Statistics for Windows, version 25 (IBM, Armonk, NY).

Ethical approval was waived by the Medical Ethics Review Committee of Erasmus MC, University Medical Center Rotterdam (reference no. 58158).

## Results

We observed 4,666 HH opportunities with 476 nurses in 36 nursing home units. Before the intervention, substitution (15% of HH opportunities) was performed more often than HH without gloves (9% of HH opportunities). After the intervention, substitution remained 15%, while HH without gloves increased from 9% to 30% (OR, 3.40; 95% CI, 2.55–4.55). There was a slight decrease in gloves that were inappropriately unchanged (13% to 9%) and a slight increase in HH with donning and doffing gloves (3% to 9%).

**Fig. 1.**
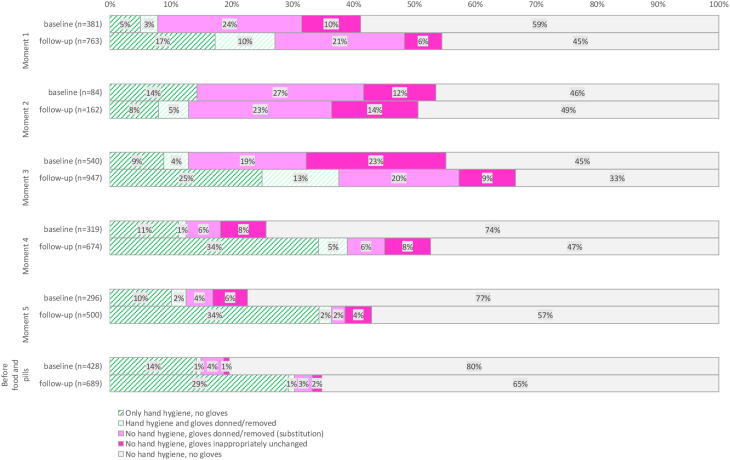
Hand hygiene compliance and glove use at the 5 WHO moments during baseline (n = 2,048 hand hygiene opportunities) and follow-up (n = 3,735 hand hygiene opportunities.

Next, we compared WHO moments at baseline versus follow-up (Fig. 1). Substitution varied per moment at baseline (4%–27%). During follow-up, we observed little change in substitution per moment compared to the baseline (0% to −4%). The combination of HH and gloves occurred infrequently at the baseline (0%–4%) and remained infrequent for most moments after the intervention (1%–13%).

Moment 3 showed the largest decrease in inappropriately unchanged gloves (−14%; OR, 0.48; 95% CI, 0.33–0.68). There was little change (−4% to +3%) in substitution between baseline and follow-up for studied submoments. There were relevant changes in inappropriately unchanged gloves for moment 3: after washing or performing perineal care in the resident’s room (−26%; OR, 0.19; 95% CI, 0.10–0.36) and after residents were helped at the toilet (−20%; OR, 0.18; 95% CI, 0.06–0.50). Correctly performing HH with gloves occurred more frequently at follow-up, specifically after helping the resident at the toilet (+22%; OR, 7.94; 95% CI, 1.72–36.59), after perineal care in the resident’s room (+17%; OR, 36.59; 95% CI, 4.87–274.90), and before washing or performing perineal care in the resident’s room (+11%; OR, 3.10; 95% CI, 1.40–6.89).

We investigated whether individual nurses’ behavior changed at follow-up (345 nurses; mean, 13 opportunities; range, 5–37; standard deviation, 6). The percentage of nurses who performed substitution at least once remained stable (Table 1). We detected a 15% increase in nurses who combined HH with glove donning and doffing at least once and a 15% decrease of nurses who inappropriately did not change gloves at least once.

**Table 1.** Individual Nurses’ Behavior During the Study^a^

Action	Baseline (n=109), %	Follow-up (n=236), %	Difference, %	Odds Ratio (95% CI)^b^
Performed hand hygiene with donning and doffing gloves at least once	28	43	+15	1.98 (1.20–3.28)
Replaced hand hygiene with gloves (substitution) at least once	73	72	−1	0.91 (0.55–1.53)
Inappropriately unchanged gloves at least once without doing hand hygiene	49	34	−15	0.50 (0.31–0.82)
Never did hand hygiene, never wore gloves	12	4	−8	0.40 (0.18–0.90)

Note. CI, confidence interval.

a 345 nurses, of whom 15% were nursing students.

b Odds ratios were corrected for the clustering of observations within nursing homes in a multilevel analysis.

## Discussion

We investigated whether an HH intervention in nursing homes changed glove usage. Substitution occurred at 15% of HH opportunities at baseline and did not decrease at follow-up. At moment 3 (ie, after body fluid exposure risk), there was a marked reduction of inappropriately unchanged gloves (−17%). There were increases in performing HH with donning and doffing gloves at 3 submoments. The percentage of nurses who performed substitution at least once remained stable.

Other studies have also reported little change in substitution after an HH interventions.^7–10^ In our study, facilities for HH were often lacking in the residents’ rooms (29% of nursing home units lacked a sink, 54% lacked ABHR), possibly explaining why substitution remained constant.

A strength of the study is that not only the WHO Moments but also the frequently occurring submoments were investigated. Furthermore, individual nurse’s behavior was analyzed. A limitation is that only nurses were observed, although nurse’s aides provide substantial care in nursing homes.

In conclusion, the intervention was not successful in reducing substitution of HH by glove use, even though the training addressed substitution.^5^ We observed significant positive changes in HH with donning and doffing gloves as well as a significant decrease in inappropriately unchanged gloves after contact with body fluids. Nurses in nursing homes need dedicated glove-use training.
